# Management of Acute Lateral Humeral Condyle Fractures in Children

**DOI:** 10.3390/children11121421

**Published:** 2024-11-25

**Authors:** Mónica Álvarez Muñoz, Juan Carlos García de la Blanca, Myriam Vidart Anchía, Rafael Martí Ciruelos, Sara Calvo Calvo, María Teresa Menéndez Crespo

**Affiliations:** Service of Traumatology and Orthopaedics Surgery, Hospital 12 de Octubre, 28041 Madrid, Spain; juanca12@ucm.es (J.C.G.d.l.B.); myriam.vidart@salud.madrid.org (M.V.A.); rafael.marti@salud.madrid.org (R.M.C.); sara.calvocal@salud.madrid.org (S.C.C.); mariateresa.menendez@salud.madrid.org (M.T.M.C.)

**Keywords:** lateral humeral condyle, pediatric, diagnosis, treatment, complications

## Abstract

Pediatric elbow fractures are quite common, accounting for up to 34% of bone fractures in children. Among these, acute lateral humeral condyle (LHC) fractures represent up to 22%. The accurate diagnosis and early treatment of LHC fractures are crucial due to the potential for abnormal growth and significant long-term impacts on joint motion. With the aim of enhancing the understanding of pediatric LHC fracture management among pediatric healthcare practitioners, we present a literature review combined with our technical recommendations based on our experience. Imaging through AP, lateral, and internal oblique X-rays remains the gold standard for diagnosis, although there is increasing focus on non-irradiating techniques, considering the skeletally immature nature of the patients. Several classification systems aid in fracture assessment, each varying in their simplicity, reproducibility, and inter- and intra-observer correlations. The treatment approaches for LHC fractures include conservative management with immobilization for minimally displaced fractures and surgical intervention for displaced fractures. The surgical options encompass closed and open reductions, using Kirschner wires or cannulated screws for fixation. While both methods show favorable outcomes, recent years have seen a growing interest in expanding the traditional indications for closed approaches. After a period of post-surgical immobilization of the limb, rehabilitation care is recommended to assist in the recovery of the range of motion. During the postoperative period, the most frequent complications are bony overgrowth, malunion, and infection, although with highly variable rates, which typically do not result in functional impairment if managed properly. Regular follow-up and monitoring are essential for optimal recovery and minimizing long-term complications.

## 1. Introduction

Up to 34% of pediatric patients experience a bone fracture, and the elbow joint ranks among the most frequent sites, with rates reaching up to 28% [1]. Due to a significant proportion of fractures being subtle and presenting minimal displacement, they can go unnoticed. However, both their diagnosis and early treatment are crucial. The skeletal immaturity of pediatric patients predisposes them to the risk of experiencing abnormal growth during development if injuries at the physis are not properly addressed. Specifically, in fractures involving an articular surface, the long-term consequences on the range of motion can be significant [2,3,4,5].

In this literature review, we aim to outline the fundamental principles of managing acute lateral humeral condyle (LHC) fractures to guide pediatric healthcare practitioners. We conducted a comprehensive bibliographic search focusing on the epidemiology, classification, diagnosis, treatment, and complications of this condition in pediatric and adolescent patients, selecting articles published in English. Due to the relative scarcity of literature on some topics, we included systematic and narrative reviews, biomechanical studies, prospective and retrospective research, and small case series. Although our initial aim was to include only articles published within the last 15 years, we expanded the scope to incorporate previous but relevant studies. Articles addressing other types of pediatric elbow fractures or published in non-indexed journals were excluded. Additionally, we shared our clinical insights and recommendations based on our experience in a tertiary hospital, integrating them into the discussions within each section.

### 1.1. Epidemiology

LHC fractures are the second most common among pediatric elbow fractures, after supracondylar fractures, accounting for up to 22% of injuries in this joint [1].

These fractures most commonly occur in children aged between 5 and 8 years, with the peak incidence observed at 6 years old, although there are cases reported in the literature of infants younger than 2 years old [1,6,7]. Many LHC fractures happen during sports practice or leisure activities in the park or school playground, and in some regions, they exhibit a higher prevalence during the summer months. There is a greater tendency to fracture the non-dominant limb, as the dominant arm is typically engaged in the activity at hand [1,8].

While isolated injuries are the most common presentation, they can also be associated with fractures of the radial head and proximal ulna, as well as with joint dislocation [1,4].

### 1.2. Mechanism of Fracture

The most common mechanism of fracture is the so-called “push-off”, in which, due to a fall with the arm outstretched in supination, a varus injury occurs with a lateral condyle avulsion fracture. Alternatively, they can also occur due to a fall on a flexed elbow or from a direct blow to the lateral side of the joint, impacting on the lateral condyle [1,4,9].

### 1.3. Classification

The accurate classification of LHC fractures can be crucial in determining the optimal treatment for each case. There are different systems that have been validated and are widely used in clinical practice, each based on various criteria. Although all are accepted, their simplicity of application, reproducibility, and guidance in treatment differ, so the latest research evidence can help in choosing among them [10].

#### 1.3.1. Fracture Pattern

The Milch classification [11] divides LHC fractures into two types based on the anatomical landmarks that the fracture trace affects.

- Type I fractures exit the joint lateral to the trochlea. They represent a Salter–Harris (physeal fracture classification) type IV fracture and are considered stable fractures.
- In type II fractures, the most prevalent type, the fracture line passes through the trochlea. They are considered a Salter–Harris type II fracture and are potentially unstable.

#### 1.3.2. Fracture Displacement and Articular Congruence

The Jakob classification [12] establishes that the fracture originates from the lateral aspect of the condyle, with three types categorized based on fragments’ displacement.

- In type 1 fractures, there is a non-displacement (<2 mm), without the fracture line reaching the joint. This leaves an intact cartilage bridge that acts as a hinge helping in the correct reduction of the fragment.
- In type 2 fractures, there is a minimal displacement of 2–4 mm, and the fracture line exits through the joint but without rotation of the fragment.
- In type 3 fractures, there is a displacement greater than 4 mm with a rotational component and a loss of radio-capitellar congruence.

The Weiss classification [13] is primarily based on fragment displacement and was developed with the aim of establishing treatment recommendations and predicting complications.

- In type 1 fractures, there is a <2 mm displacement, with an intact articular surface. Conservative management is recommended.
- In type 2 fractures, there is a ≥2 mm displacement, maintaining the intact articular surface. Closed reduction and pinning are recommended.
- In type 3 fractures, there is a ≥2 mm displacement and loss of articular congruence. Open reduction and internal fixation are recommended.

#### 1.3.3. Fracture Pattern, Displacement, and Stability

The Song classification [14] combines the assessment of fragment displacement, fracture pattern, and stability, thus incorporating criteria used by other systems and making it the most comprehensive one.

- In Stage 1, there is a displacement of ≤2 mm, with the fracture line located in the metaphysis and stable; all four radiographic views are necessary for assessment.
- In Stage 2, there is a displacement of ≤2 mm, and the fracture line extends to the articular cartilage of the epiphysis with a small lateral gap. Its stability is indeterminate, and all four radiographic views are necessary for assessment.
- In Stage 3, there is a displacement of ≤2 mm, with a gap extending medially and laterally. The fracture is unstable and observable in any of the radiographic views.
- In Stage 4, there is a displacement of >2 mm, with intra-articular fracture, without rotation of the fragment. The fracture is unstable and observable in any of the radiographic views.
- In Stage 5, there is a displacement of >2 mm, with intra-articular fracture, with rotation of the fragment. The fracture is unstable and observable in any of the radiographic views.

#### 1.3.4. Choosing the Classification System

Currently, there is no consensus on the recommended classification system for LHC fractures in clinical practice. However, individual recommendations have been published based on inter- and intra-observer correlations, which were found to be lower in the Milch classification compared to the Weiss classification [15], with acceptable values in the Jakob classification—though lower for fractures with a rotational component [16,17]—and high values in the Song classification. The latter, however, can pose a challenge in distinguishing between subtypes 2–4 [10]. We believe the choice of a classification system should ultimately depend on the specialist, the hospital setting, and the specific clinical practices of the center, using the selected systems in all patients to ensure uniformity. In our practice, we generally follow the Jakob and Milch classifications, while acknowledging the broader applicability of the Song classification in a scientific context.

## 2. Diagnosis

### 2.1. Physical Examination

Most patients present with localized pain and swelling on the lateral aspect of the distal humerus, along with functional impairment manifested as a limited range of motion in the upper limb. During the initial medical examination, it is crucial to assess the neurovascular status of the elbow joint, checking for the presence of hematomas, which may indicate fracture displacement and instability [18].

### 2.2. Imaging Tests

#### 2.2.1. X-Rays

**Figure 1:**
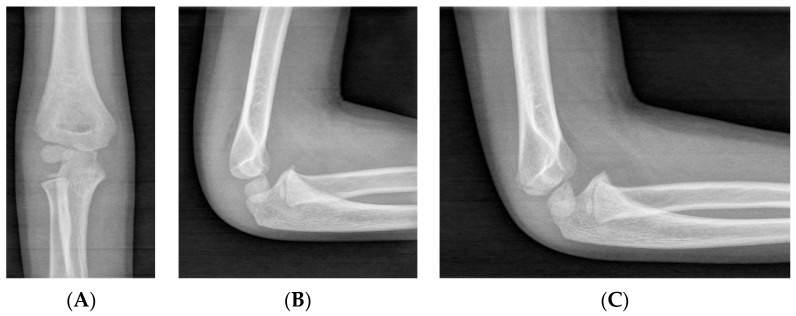
Non-displaced LHC fracture, for which conservative treatment is recommended. (**A**) An AP view, where the fracture is vaguely visible. (**B**) A lateral view, where the fracture is not appreciable. (**C**) An internal oblique view, where the characteristics of the fracture are clearly distinguishable.

Currently, X-ray remains the diagnostic gold standard for pediatric LHC fractures, with general recommendations including obtaining an anteroposterior (AP) view and a lateral view. While some specialists suggest that an additional internal oblique radiograph should be used in cases of uncertainty, others recommend its routine use, as it can provide a clearer view of fracture displacement (Figure 1). This issue should be emphasized to residents and young surgeons, highlighting the need for a high index of suspicion of an LHC fracture when encountering a patient with elbow pain and functional impairment. If necessary, comparison with an X-ray of the contralateral elbow can be performed [19,20,21].

Radiographic diagnosis can be challenging due to factors related to the age of the patients. Pediatric patients in pain may not fully cooperate during imaging, while the incompletely ossified epiphysis can complicate fracture identification, especially when there is no or minimal displacement [19,20,21].

#### 2.2.2. Ultrasonography

Ultrasonography offers the advantage of assessing the state of the articular cartilage, cortical disruption, and differentiation between stable and unstable fractures. With a sensitivity ranging from 92.9% to 98% in pediatric elbow fractures, it provides an effective and rapid diagnostic alternative, as it is readily available in emergency settings [19,22,23].

However, its limitation lies in its dependency on the skills of the sonographer. While it cannot currently replace radiography entirely, it remains a valuable diagnostic tool that avoids radiation exposure for patients [19,22].

#### 2.2.3. Magnetic Resonance Imaging (MRI)

MRI provides a high-resolution visualization of soft tissues, a particularly valuable capability in this context as it allows for the observation of the incompletely ossified epiphysis. It proves especially useful in patients with undisplaced or minimally displaced fractures, aiding in the assessment of articular cartilage integrity and the classification of fractures as stable or potentially unstable [24,25].

However, its drawbacks include its limited availability, the necessity for sedation in patients under 6–7 years of age, and its high cost. Therefore, our recommendation is to reserve MRI for complex cases.

#### 2.2.4. Computed Tomography (CT)

CT has emerged as a valuable tool for determining fracture classification and displacement, albeit with the drawback of high radiation exposure. However, the advent of multidetector CT (MDCT) scanners in many centers offers the potential to minimize the radiation dose to the elbow joint. This advanced technology enables a rapid evaluation of cartilage damage through a painless procedure, eliminating the need for patient sedation [26].

CT assessment is particularly advantageous in fractures where the displacement may be challenging to ascertain with other imaging modalities, especially in cases where treatment decisions hinge on evaluating such displacement (e.g., fractures with displaced fragments close to 2 mm) [26]. Nevertheless, its utilization remains limited, and like MRI, it is reserved for cases that are difficult to examine using more conventional diagnostic methods.

#### 2.2.5. Arthrography

Arthrography, while offering a direct visualization of intra-articular structures, is experiencing declining diagnostic usage. Its invasive nature prompts a preference for MRI or CT when a detailed soft tissue evaluation is required. Nonetheless, it remains a widely used intraoperative method to assess articular cartilage congruency [7,27].

#### 2.2.6. Choosing the Imaging Test

The anatomical characteristics of pediatric patients require a careful consideration of radiation exposure. Therefore, considering the complexity of each case, a balance must be struck between maximizing diagnostic accuracy and minimizing exposure, for which the previously described alternatives can be utilized [19].

**Figure 2:**
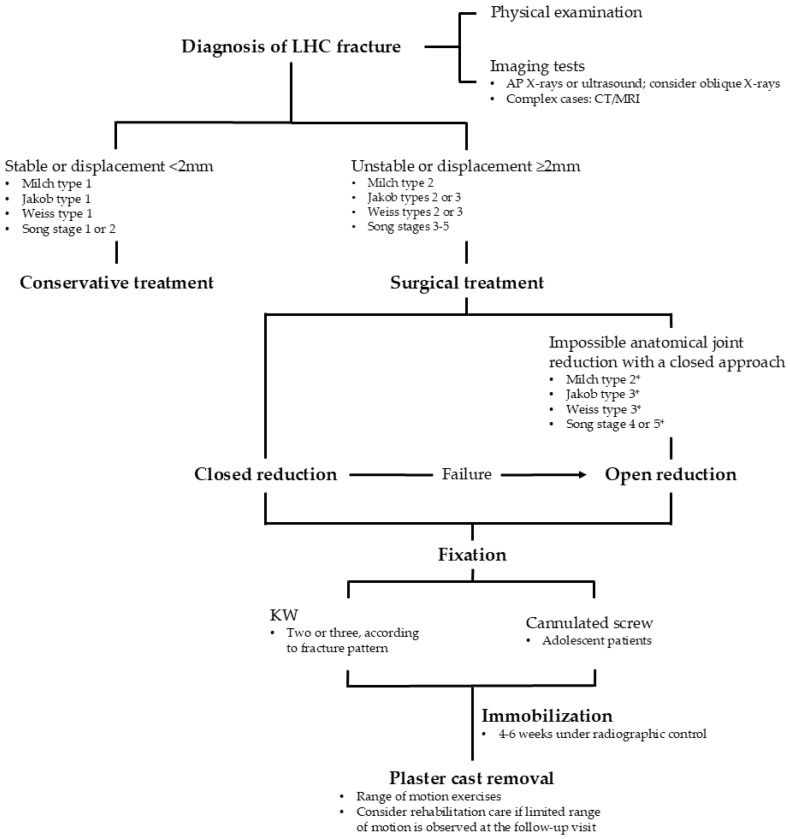
Diagnostic and treatment algorithm for pediatric LHC fractures at our hospital. * Indicates possible cases that may be unmanageable with closed reduction, although in practice many can be successfully treated with a closed approach.

That said, we are mindful of the practical challenges we face in clinical settings, including costs, equipment availability, and time constraints. Therefore, in our practice, we rely primarily on anteroposterior (AP) and lateral X-rays for diagnosis, reserving internal oblique views for cases where there is diagnostic uncertainty in order to minimize patient radiation exposure. Specifically, we recommend the use of internal oblique views in non-displaced or minimally displaced fractures, as this projection provides a better visualization of the true fragment displacement, aiding in the decision between conservative and surgical treatment (Figure 2). In more complex cases, we conduct an individual assessment of diagnostic needs to determine which imaging test we will use.

## 3. Treatment

Currently, the management of LHC fractures in pediatric patients remains contentious, primarily revolving around identifying stable fractures with a minimal risk of secondary displacement. Notably, a considerable percentage (10–45%) of initially undisplaced fractures may eventually become displaced, underscoring the importance of vigilant monitoring and explaining why some authors advocate for surgical intervention in all LHC fractures to pre-empt potential complications [28,29,30].

### 3.1. Conservative Treatment

Conservative treatment, involving immobilization with long-arm casting, is widely endorsed for undisplaced fractures or those with a displacement of <2 mm [29,31]. In our clinical practice, we tend to use a brachiopalmar splint in supination and wrist extension, as we believe it offers greater comfort and better meets the needs of pediatric patients.

The recommended duration of immobilization varies in the literature, ranging from 3 to 8 weeks. It is advisable to conduct periodic checks to monitor secondary displacement. Studies usually recommend the first follow-up examination between 3 and 7 days post-fracture, followed by weekly check-ups until consolidation [31,32].

### 3.2. Surgical Treatment

For fractures with >2 mm displacement, there is a consensus favoring surgical treatment [7,30,33].

Following the fundamental principle of fracture management, all surgical interventions aim to achieve reduction, restoration of the anatomical joint alignment, and fixation using instrumentation to stabilize the physis and prevent displacement. Various surgical approaches are available depending on the type of reduction and fixation methods employed [31].

#### 3.2.1. Closed Reduction

Traditionally, closed reduction has been recommended for stable fractures with displacement between 2 and 4 mm, while more unstable fractures may carry a higher risk of nonunion when treated with this approach [27,31].

The closed reduction procedure involves applying a varus force to the elbow while maintaining the forearm in supination, followed by manual repositioning of the fragment into its anatomical location [7]. If this maneuver is unsuccessful, a Kirschner wire (KW) can be inserted into the condyle and used as a joystick to facilitate reduction [4]. Before proceeding with fixation, it is crucial to ensure that anatomical alignment has been achieved. Since it cannot be assessed in situ—which is considered the main drawback of the technique—various imaging methods must be used [1,4,31].

Following closed reduction maneuvers, confirmation of correct joint congruence is typically achieved through X-rays or arthrography [7,14,27]. Recent studies have explored alternative imaging modalities for verification. Li et al. (2021) utilized ultrasonography to assess fracture reduction, reporting satisfactory outcomes in a series of 42 patients [34]. Similarly, Xie et al. (2020) performed closed reduction followed by radiographic and arthrographic evaluations in 46 patients [7], and Kang et al. (2019) employed arthroscopy to aid in closed reduction for 39 patients, noting its safety but highlighting a learning curve of approximately 6 months and 12 cases for this technique [7].

In case of reduction failure, or if success cannot be determined, conversion to open reduction is recommended [35].

#### 3.2.2. Open Reduction

Open reduction is the preferred approach for many pediatric surgeons to achieve anatomical reduction in fractures with >4 mm fragment displacement, those deemed unstable, or cases involving disrupted articular surfaces or rotated fragments. It also serves as a rescue alternative when closed reduction is ineffective [1,7].

The primary advantage of open reduction lies in the exposure and direct visualization of the fracture, facilitating an in situ assessment of the reduction. Fragment fixation prevents secondary displacement and leads to lower rates of malunion compared to closed reduction [7]. However, this is an invasive procedure, requiring meticulous attention to avoid complications such as AVN, which can arise from tissue dissection [1,4,7].

The lateral (Kocher) approach is commonly used and is our preferred technique, with a working window between the anconeus, innervated by the radial nerve, and the extensor carpi ulnaris, innervated by the posterior interosseous nerve. In cases of fractures with significant displacement, the fragment has already created an intermuscular dissection. Therefore, we utilize this existing pathway, eliminating the need for an additional incision. Vascularization of the condyle occurs through its posterolateral portion, making it vital to avoid periosteal stripping in this area [4]. During the procedure, it is essential to directly observe the articular line. Once the reduction is performed, the surgeon should verify the anatomical alignment and assess the range of elbow flexion–extension [31,35].

#### 3.2.3. Choosing the Reduction Method

The traditional recommendations followed by many specialists—closed reduction for minimally displaced stable fractures and open reduction for more complex fractures—continue to guide clinical practice. However, a significant percentage of surgeons opt to attempt closed reduction initially, resorting to open reduction if necessary [7,14,27], while others consistently employ open reductions, even for minimally displaced fractures, as it is easier to ensure anatomical reconstruction [30,33,36].

In recent years, there has been growing interest in minimally invasive procedures across all surgical fields, and the management of pediatric LHC fractures is no exception. Through various studies, Song et al. (2008) demonstrated the effectiveness of closed reduction in all fracture types, progressively achieving more favorable outcomes due to the learning curve associated with the procedure [14,31,37].

When comparing open and closed reduction for fractures with a displacement of >4 mm, Xie et al. (2021) found no significant differences in terms of complications or prognosis between the two approaches [27], and Liu et al. (2022) reported a significantly higher rate of excellent clinical outcomes with closed reduction [38]. Similar findings were reported in studies by Ganeshalingam et al. (2018) [33], who observed a—not statistically significant—6% higher rate of complications in patients treated with closed reduction, and by Koh et al. (2010) [39], who instead noted a significantly higher incidence of lateral overgrowth and osteophyte development in patients undergoing open reduction, but with no clinical impact.

In summary, while achieving similar outcomes, open reduction may pose risks that outweigh its benefits for fractures amenable to closed reduction. Given its lesser invasiveness, our personal recommendation is that the closed approach should be considered first, provided that the surgeon is confident in achieving effective anatomical joint reduction. In cases where it is not feasible, open reduction should be chosen (Figure 2). Regardless of the reduction method employed, subsequent fixation can be accomplished using a KW or cannulated screws [31].

#### 3.2.4. Fixation: Kirschner Wires

**Figure 3:**
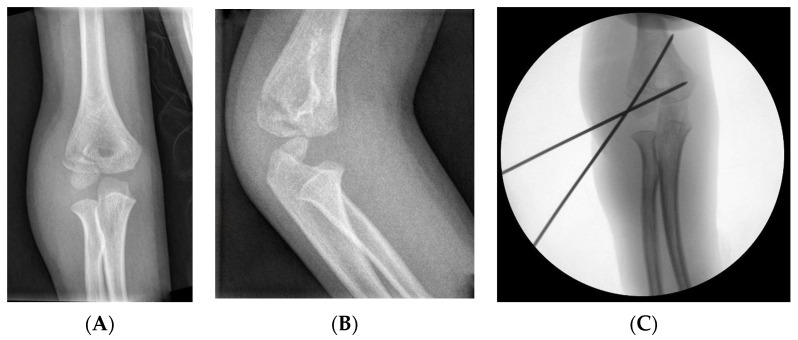
LHC fracture with 2 mm displacement. (**A**) AP view. (**B**) Internal oblique view. (**C**) Treatment with KWs, AP view.

This is the fixation method preferred by most authors, using two or three KWs in a parallel or divergent position and passing through the contralateral cortex without crossing at the fracture site [4,6,31,36,39,40] (Figure 3). Depending on the fracture pattern, a KW parallel to the joint and/or a KW on the medial side can be used. In the case of open reductions, they must be inserted posteriorly to the incision [35].

KWs can be placed percutaneously, which simplifies removal [31,33,36], or subcutaneously [6], and they are usually removed after 4–6 weeks. However, shorter periods of 2–3 weeks have also been reported to provide good results [41].

#### 3.2.5. Fixation: Cannulated Screws

**Figure 4:**
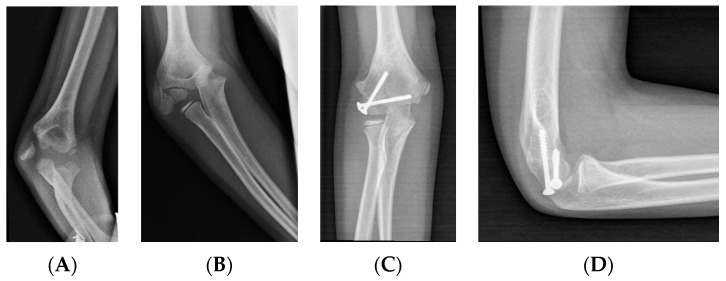
LHC fracture with >2 mm displacement and rotated fragment. (**A**) AP view. (**B**) Lateral view. (**C**) Treatment with two cannulated compression screws, AP view. (**D**) Treatment with two cannulated compression screws, lateral view.

While K-wires allow for limited compression, cannulated screws offer a more favorable alternative in this regard and can be used in patients with a sufficiently long metaphyseal fragment [31] (Figure 4).

During closed reductions, the screw is inserted percutaneously, while in open reductions, it can also be inserted through the incision. Stein et al. (2017) argued in their work that the use of cannulated screws with closed reduction and percutaneous synthesis offers good results and avoids the complications of the open approach [42].

#### 3.2.6. Choosing the Fixation Strategy

Li et al. (2012) studied 62 patients treated with KWs and cannulated screws and concluded that both methods have their advantages: KWs can pass through the ossification nucleus without damaging it and are easy to remove. In contrast, cannulated screws reduce the incidence of lateral overgrowth and provide more stable fracture fixation [43]. However, cannulated screws require a second surgery for removal, around 6 months after their insertion [31,35]. Conversely, Wendling-Kleim et al. (2021) compared outcomes in 43 patients treated with KWs to those in patients treated with cannulated screws, finding better results in terms of the range of motion and pseudarthrosis among the KW-treated group [30].

While biomechanical studies have suggested that screws provide more stability than KW pinning, the pediatric lateral condyle typically permits the insertion of only a single screw, which can be reserved for adolescent patients [31,44]. Some authors have suggested that combining screws with KWs is optimal to ensure the rotational stability of the fragment [1,30].

In recent years, new fixation methods using bioabsorbable materials have been sought, with promising clinical results. While these materials eliminate the need for a second procedure to remove the implants, their higher costs limit their availability in all clinical centers [45].

Generally, the preferred fixation strategy is to insert a KW or screw—if the patient is an adolescent—parallel to the joint, with a second KW or screw placed through the lateral pillar. Alternatively, we use two slightly divergent KWs or screws—also in adolescent patients—through the lateral pillar. The decision between these approaches, as well as the use of a third KW to provide additional support in complex fractures, is based on the goal of achieving maximum stability according to the fracture pattern [46].

Regardless of the fixation method, the patient’s forearm is often immobilized with a plaster cast for a period ranging from four to six weeks, depending on the patient’s signs of bone healing observed in radiographs taken at two, four, and—if not yet consolidated—six weeks (Figure 2) [1].

### 3.3. Rehabilitation Care

After plaster cast removal, patients are instructed to perform flexion–extension and pronation–supination exercises at home to gradually restore mobility. Two to three weeks later, they attend a follow-up to assess their range of motion, and if their mobility is deemed insufficient, they are referred to a hospital-based rehabilitation program.

During the first month post-treatment, patients are advised to avoid all sports activities. After the second month, low-impact sports, such as swimming, are permitted, but contact sports should be avoided for four to six months.

## 4. Complications

The incidence of complications in pediatric LHC fractures is higher than that in other elbow fractures, with up to 10% of patients experiencing events that significantly impact their quality of life, which may arise during or after treatment [1].

### 4.1. Bony Overgrowth and Deformity

Lateral overgrowth, defined as an increase in the interepicondylar width with no signs of angular deformity, is a prevalent complication observed in 70–100% of patients. Some specialists even consider the development of a lateral spur as an expected outcome, especially in surgically treated displaced fractures [7,31,39,47].

Due to their clinical and radiographical similarities, lateral overgrowth can be confused with cubitus varus during the initial examination. However, cubitus varus is defined as an increase in the Baumann angle of greater than 5° compared to the contralateral side [47], with a lower incidence ranging from 29 to 40% [4,47].

While neither condition typically results in diminished elbow function or necessitates surgical intervention, vigilant monitoring of cubitus varus is advisable, as select cases may require corrective osteotomy [4,39,47].

Other less common deformities include cubitus valgus, ulnar valgus, and fishtail deformity [4,27,33].

### 4.2. Nonunion

Nonunion is a significant concern among pediatric elbow fractures, particularly in cases of LHC fractures, where the prevalence rates vary widely across studies due to the absence of standardized temporal criteria for its definition. While some authors describe nonunion as the absence of consolidation within 8 weeks, others classify it as delayed union during this timeframe and reserve the term nonunion for cases persisting at 12 weeks [4,41,48].

Nonunion can arise from fixation method failure, from secondary fragment displacement in conservatively managed fractures, or as a delayed presentation. While more prevalent in conservatively treated cases, it has also been observed in surgically managed patients [33,48,49].

The treatment options for nonunion include closed or open reduction and synthesis [48]. In a meta-analysis by Zhang et al. (2022), percutaneous fixation appeared to be associated with inferior outcomes compared to open reduction and internal fixation, although it may result in improved mobility rates [50].

### 4.3. Malunion

Malunion differs from nonunion in that it involves fracture healing but fails to maintain the natural anatomical alignment, resulting in an abnormal joint shape that can lead to elbow pain and stiffness.

Its incidence can reach up to 12%, and if left untreated—usually through an intra-articular osteotomy—it can result in aforementioned deformities, such as cubitus varus and valgus [1].

### 4.4. Avascular Necrosis

AVN is one of the most feared complications due to its devastating long-term implications, causing disability in patients by affecting the joint’s range of motion and functionality. Fortunately, its incidence is relatively rare, between 1% and 3% [33,39].

While open reduction poses a risk of AVN, and surgeons must be careful not to strip the posterior soft tissue, the complication has been reported in conservatively managed fractures as well, most likely due to the trauma causing the injury [1,33,39].

### 4.5. Infection

Infections are a relatively uncommon complication (0–8%) following the treatment of pediatric LHC fractures and can be categorized as either superficial or deep. Superficial infections typically respond well to hardware removal and treatment with oral antibiotics [27,33,39,48]. Deep infections are less common, tend to appear later, and, if poorly managed, can lead to severe complications, including recurrent infections. The treatment varies depending on the case, but irrigation and debridement, along with appropriate antibiotic therapy based on culture results, should be considered [51].

Both the incidence of infection and the requirement for additional surgery to remove the material are slightly higher in cases treated with KW fixation [33].

### 4.6. Loss of Range of Motion

The loss of terminal degrees of extension and flexion is common, but it typically does not result in a functional deficit [4]. However, in extreme cases where the movement restriction exceeds 20°, capsulolysis has been used as a solution [6].

## 5. Limitations

The major limitation of our study is that it is a literature review, meaning that although we conducted a comprehensive search, it was not exhaustive. Consequently, not all the studies regarding this topic have been included, and not all of the referenced studies provide primary evidence. Moreover, there is a certain degree of heterogeneity among them, as our sources include various study and review designs. We have attempted to mitigate this by supplementing the evidence with our own technical recommendations based on clinical experience. Nevertheless, our suggestions may carry some bias, as they are influenced by the clinical guidelines, resources, and logistics specific to our hospital. That said, Hospital 12 de Octubre is a tertiary referral center in Spain, where protocols are regularly updated, lending confidence to the relevance of our recommendations. Finally, generalizing recommendations in pediatric fractures can be challenging due to the unique complexities these patients present. We aimed to establish key recommendations throughout the manuscript, offering adaptable alternatives for practitioners managing more challenging cases. We believe that multicentric prospective studies are essential to develop standardized and practical clinical guidelines.

## 6. Conclusions

Acute LHC fractures in children are common and can present challenges in both diagnosis and treatment. Obtaining internal oblique X-rays can aid in diagnosing complex cases, and the choice of classification system for the fractures ultimately depends on the specialist. Fractures with displacement of <2 mm can be managed conservatively with immobilization and regular monitoring. However, displaced fractures of >2 mm often require surgical intervention. Both closed and open reduction techniques, along with fixation using KWs or cannulated screws, have shown favorable outcomes, with no clear superiority of one method over the other. Nevertheless, there seems to be a growing interest in expanding the indications for closed reduction. Overall, the associated complications usually do not lead to significant functional impairment but warrant routine monitoring throughout the treatment process to prevent sequelae.
